# Supplementary material for: Variations in the poly-histidine repeat motif of HOXA1 contribute to bicuspid aortic valve in mouse and zebrafish
Source: Nat Commun. 2023 Mar 20;14:1543. doi: 10.1038/s41467-023-37110-x (PMC10027860; doi:10.1038/s41467-023-37110-x)
Supplement: Supplementary file 1 — Supplementary Information [file 41467_2023_37110_MOESM1_ESM.pdf]

SUPPLEMENTARY INFORMATION

Variations in the poly-histidine repeat motif of HOXA1 contribute to bicuspid aortic valve in mouse and zebrafish

Gaëlle Odelin[1,*], Adèle Faucherre[2,*], Damien Marchese[3,*], Amélie Pinard[1], Hager Jaouadi[1], Solena Le Scouarnec[4], FranceGenRef Consortium[5], Raphaël Chiarelli[3], Younes Achouri[6], Emilie Faure[1], Marine Herbane[1], Alexis Théron[1,7], Jean-François Avierinos[1,8], Chris Jopling[2], Gwenaëlle Collod-Béroud[1], René Rezsohazy[3], Stéphane Zaffran[1,§]

[1]Aix Marseille Univ, INSERM, MMG, U1251, 13005 Marseille, France.
[2]Institute of Functional Genomics, University of Montpellier, CNRS, INSERM, Montpellier, France.
[3]Animal Molecular and Cellular Biology group, Louvain Institute of Biomolecular Science and Technology, Université catholique de Louvain, 5 (L7.07.10) place Croix du Sud, 1348 Louvain-la-Neuve, Belgium.
[4]l'institut du thorax, INSERM, CNRS, UNIV Nantes, 44007, Nantes, France.
[5]LABEX GENMED, Centre National de Recherche en Génomique Humaine, Evry, Paris, France.
[6]Transgenesis Platform, de Duve Institute, Université Catholique de Louvain, 1200 Brussels, Belgium.
[7]Service de Chirurgie Cardiaque, AP-HM, Hôpital de la Timone, 13005, Marseille, France.
[8]Service de Cardiologie, AP-HM, Hôpital de la Timone, 13005, Marseille, France.

* These authors contribute equally.

§ Correspondence to: Stéphane Zaffran - stephane.zaffran@univ-amu.fr
Aix Marseille Université, INSERM, MMG U1251, Faculté de Médecine, 27 Bd Jean Moulin, 13005 Marseille, France.

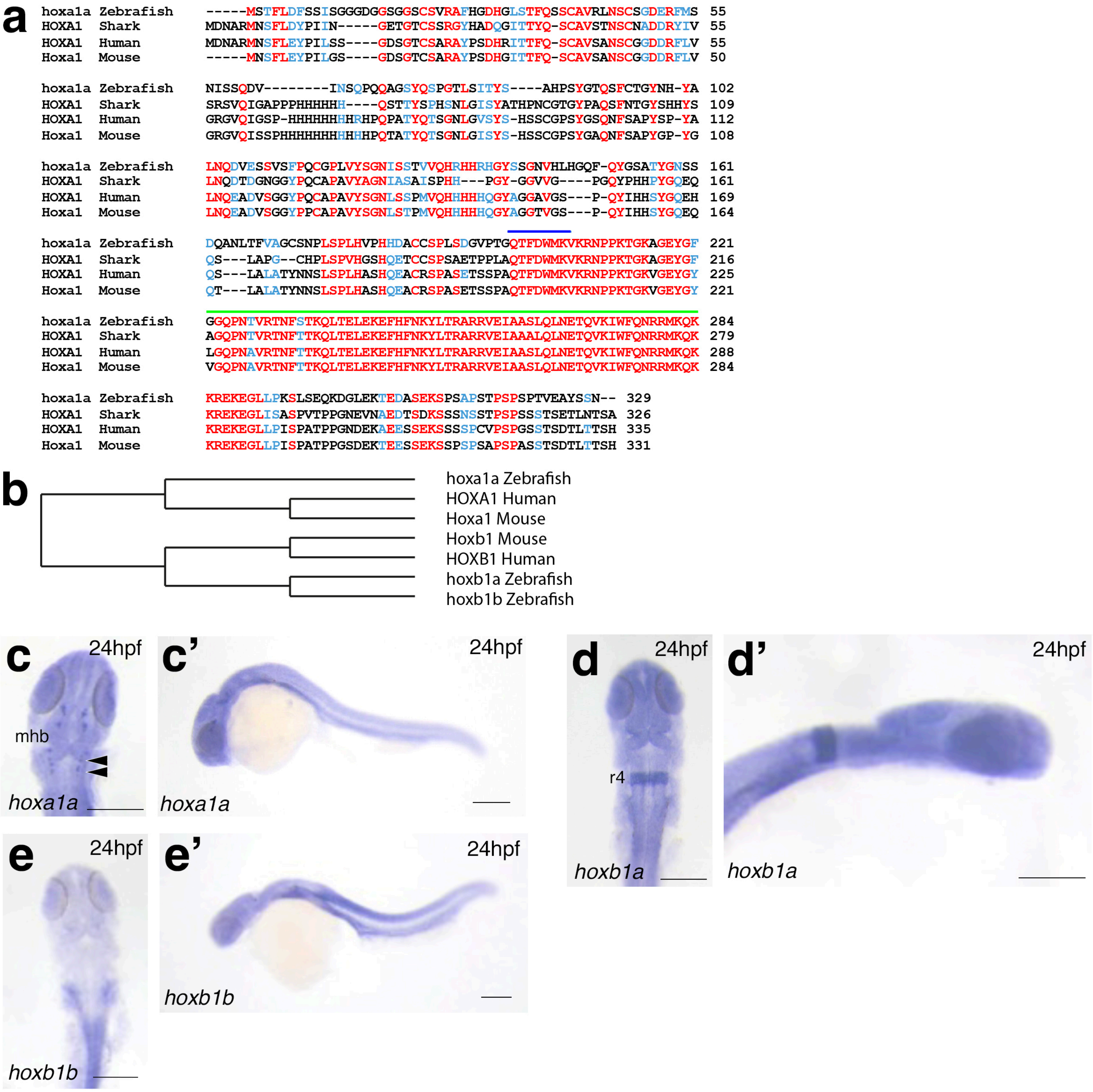

Supplementary Figure 1: Conservation of Hoxa1 ortholog and paralogs. **a**, Amino acid alignment of sequence from zebrafish (Q98SI1), shark (C7B9D2), human (P49639) and mouse (P09022) Hoxa1 orthologs. Identical residues in red, conserved changes in blue. Green line shows the homeodomain and blue line underlines the hexapeptide motif. **b**, Neighbor-joining tree showing phylogenetic relationships between Hoxa1 and Hoxb1 paralog amino acid sequences. As expected Hoxb1 provides an outgroup to all Hoxa1 sequences. **c-e**, Whole-mount in situ hybridization analysis of zebrafish *hoxa1a* (**c**), *hoxb1a* (**d**) and *hoxb1b* (**e**) genes. **c**, At 24 h post-fertilization (24 hpf) *hoxa1a* expression is localized to discrete bilateral clusters of cells in the anterior hindbrain and ventral midbrain. **d**, At the equivalent stage, *hoxb1a* is at the equivalent stage is expressed in rhombomere 4 (r4). **e**, *hoxb1b* expression is already retreated posteriorly in an anterior subpopulation of neurons. mhb, midbrain/hindbrain boundary. Data are representative of 3 experiments. Scale bars: 100 µm.

a

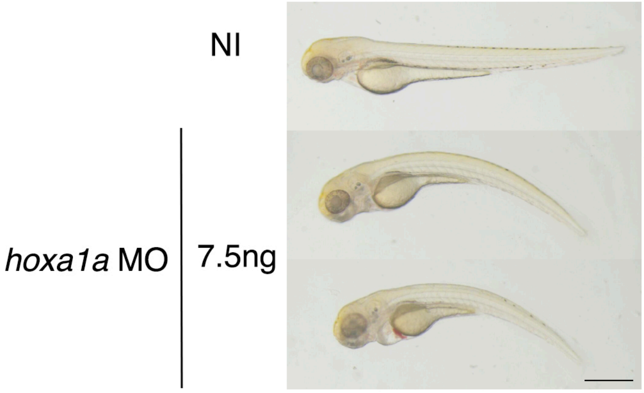

b

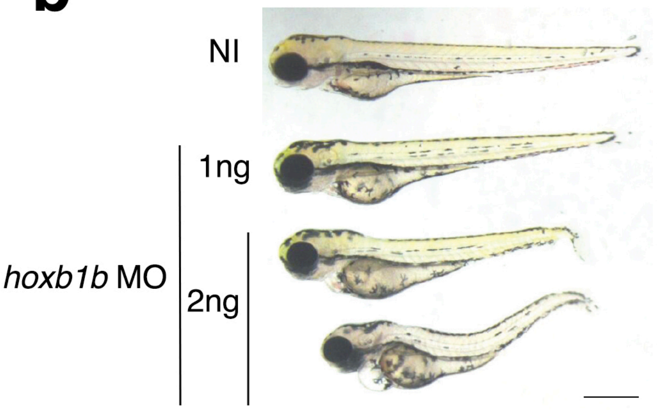

Supplementary Figure 2: Targeting *hoxa1a* and *hoxb1a* alleles. **a**, One-cell stage zebrafish embryos were injected with 7.5 ng of *hoxa1a* morpholino (MO) and examined at 72 h post-fertilization (hpf) in comparison to non-injected controls (NI). **b**, One-cell stage zebrafish embryos were injected with 1 ng and 2 ng of *hoxb1a* MO morpholino (MO) and examined at 72 hpf in comparison to non-injected controls (NI). Data are representative of 3 experiments. Scale bars: 500 μm.

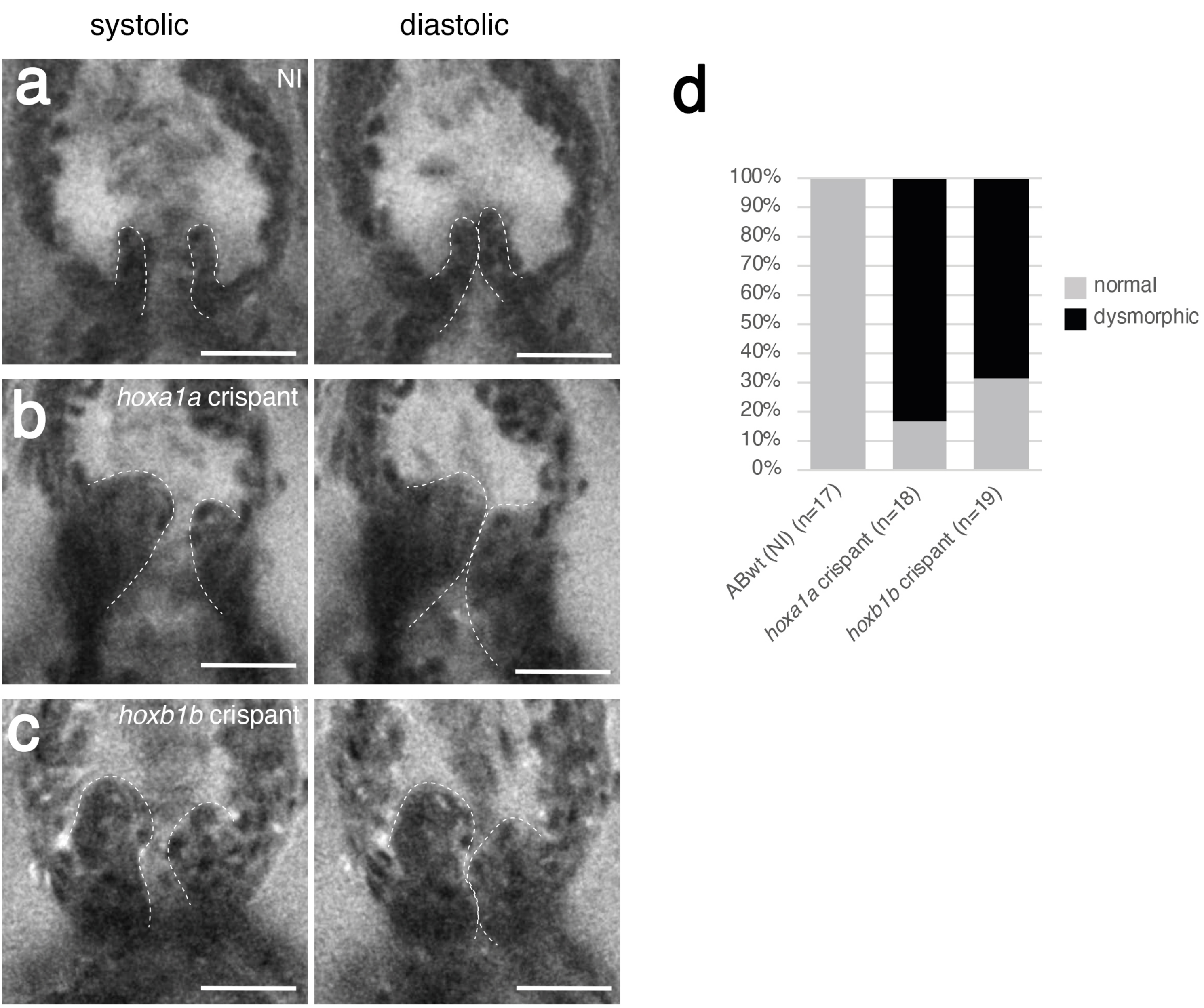

Supplementary Figure 3: Injection of gRNAs targeting either *hoxa1a* and *hoxb1a* disrupts aortic valve development. Two-photon images of aortic valves in 7 days post-fertilization (dpf) zebrafish larvae labelled with BODIPY. **a**, Representative images of aortic valve leaflets in a wild-type, non-injected larvae (ABwt, NI), valves are outlined with a dashed white line (n=17 independent embryos). **b**, gRNAs targeting *hoxa1a* (crispant) were injected into single cell zebrafish embryos (n=18 independent embryos). *hoxa1a* crispants show hypertrophic aortic valve. **c**, gRNAs targeting *hoxb1b* (crispant) were injected into single cell zebrafish embryos (n=19 independent embryos). *hoxb1b* crispants show hypertrophic aortic valve. **d**, Graph showing quantification of aortic valve defects observed in a 7 dpf larvae after injection of gRNA to knock-out *hoxa1a* or *hoxb1b* genes. Approximatively 80% of *hoxa1a* and 70% of *hoxb1a* crispants exhibited aortic valve defects. Scale bars: 20 μm. Source data are provided as a Source Data file.

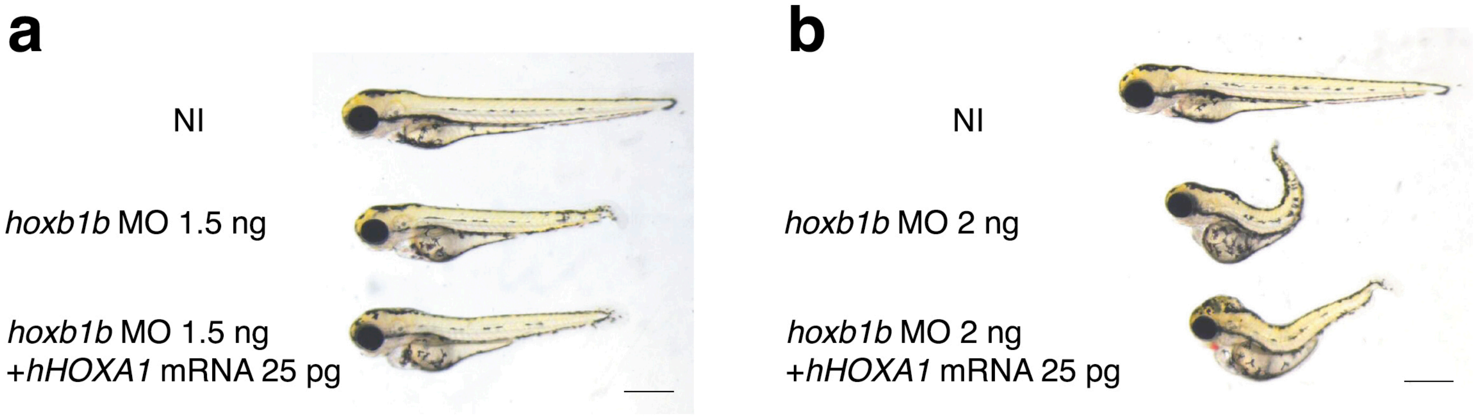

Supplementary Figure 4: Rescue experiment of *hoxb1b* morpholino. **a-b**, One-cell stage zebrafish embryos were injected with either 1.5 ng (**a**) or 2 ng (**b**) of *hoxb1b* MO alone or in combination with human wild-type *HOXA1* mRNA at 25 pg, and examined at 72 h post-fertilization (hpf) in comparison to non-injected controls (NI) Injection of human wild-type (wt) *HOXA1* mRNA cannot rescue the mild (**a**) or strong (**b**) phenotype induced by *hoxb1b* MO. Data are representative of 3 experiments. Scale bars: 500 μm.

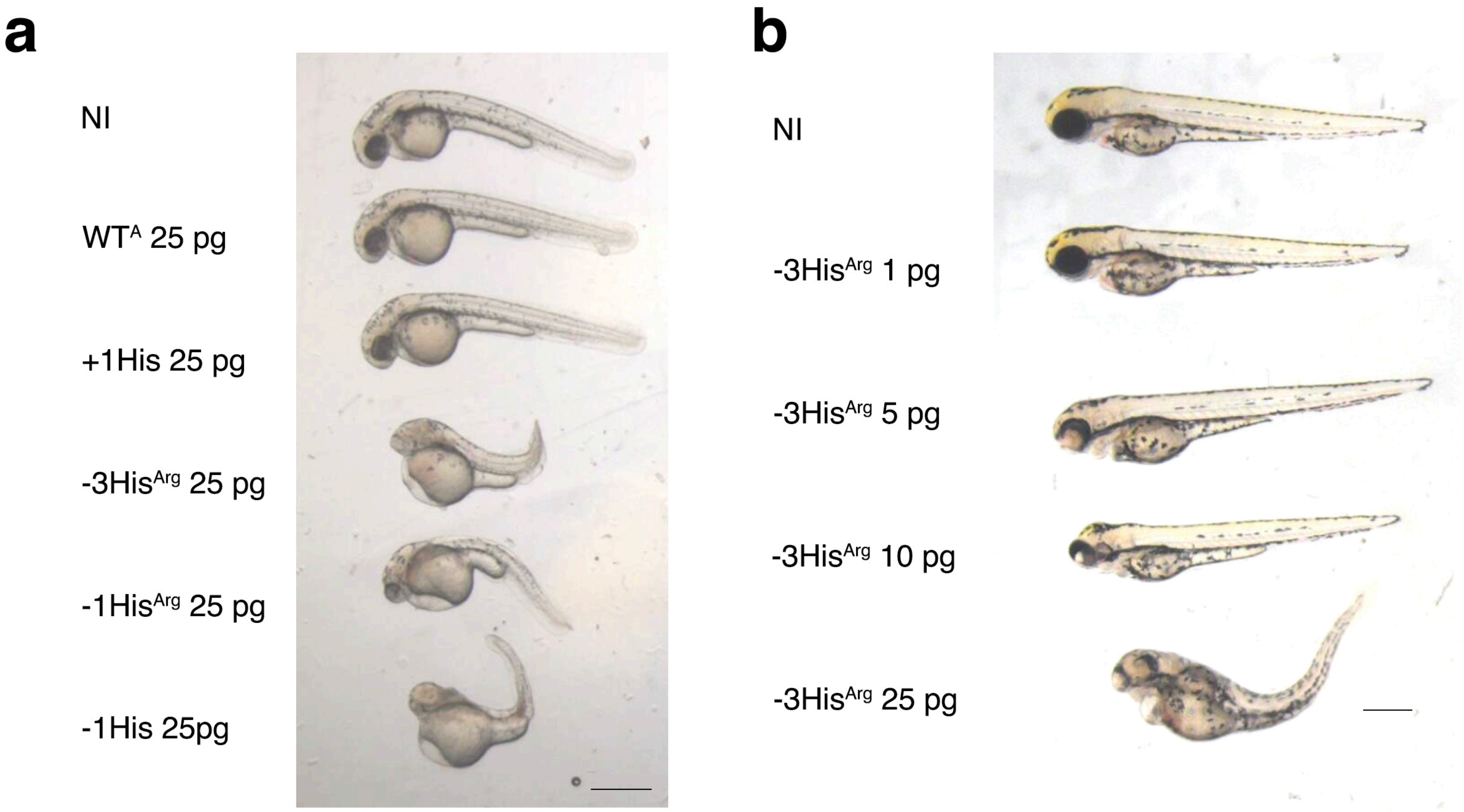

Supplementary Figure 5: Titration experiment of *HOXA1* variants. a, One-cell stage zebrafish embryos were injected with 25 pg of *HOXA1* wild-type (WT) or mutated (+1His, -1His, -1His^{Arg}, -3His^{Arg}), and examined at 48 hpf in comparison to non-injected controls (NI). **b**, One-cell stage zebrafish embryos were injected with 1 pg, 5 pg or 10 pg of *HOXA1* -3His^{Arg}, in order to determine the amount of mRNA to use for valve leaflet observation and examined at 72 hpf. Injecting 5 pg gave a phenotype mild enough to carry the experiment at 7 dpf as presented in Figure 3. Data are representative of 3 experiments. Scale bars: 500 μm.

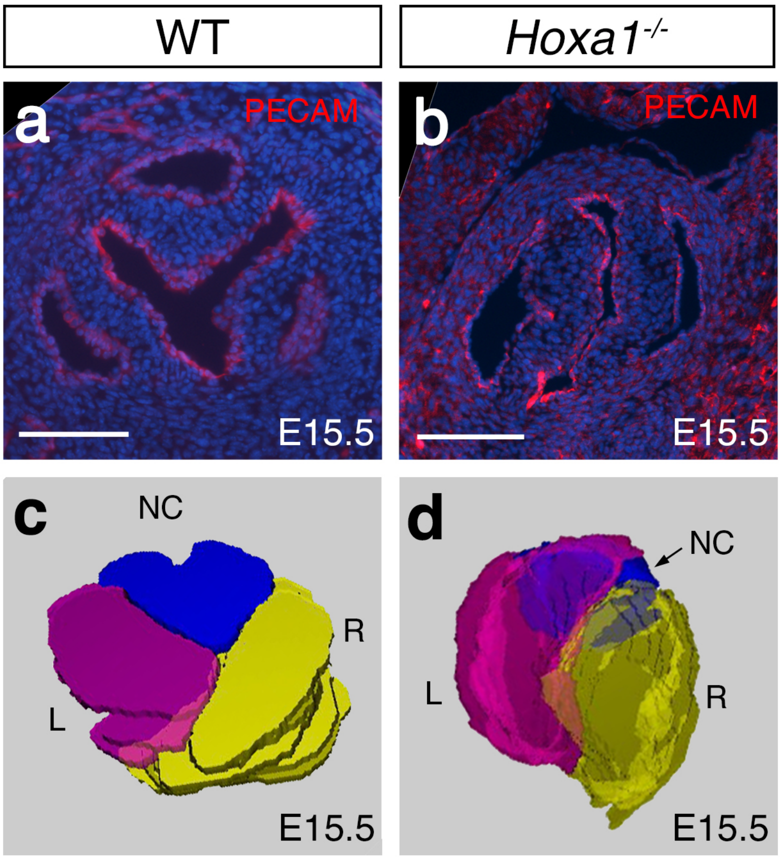

Supplementary Figure 6: Morphology of the aortic valve of *Hoxa1*$^{-/-}$ embryos at E15.5. a-b, Transversal section of E15.5 hearts immunostained with an endothelial marker, anti-Pecam antibody (red), and stained for nuclei marker (DAPI, blue). Note the bicuspid aortic valve in *Hoxa1*$^{-/-}$ (**b**). **c-d**, 3D reconstruction of histological images at E15.5 showing three aortic valve leaflets in the wild-type (WT) embryo (**c**), whereas a persistent small non-coronary leaflet is seen in *Hoxa1*$^{-/-}$ embryo (**d**). Left coronary (L; pink), right coronary (R; yellow), non-coronary (NC; blue) leaflets. Data are representative of 3 experiments. Scale bars: 100 µm (**a,b**).

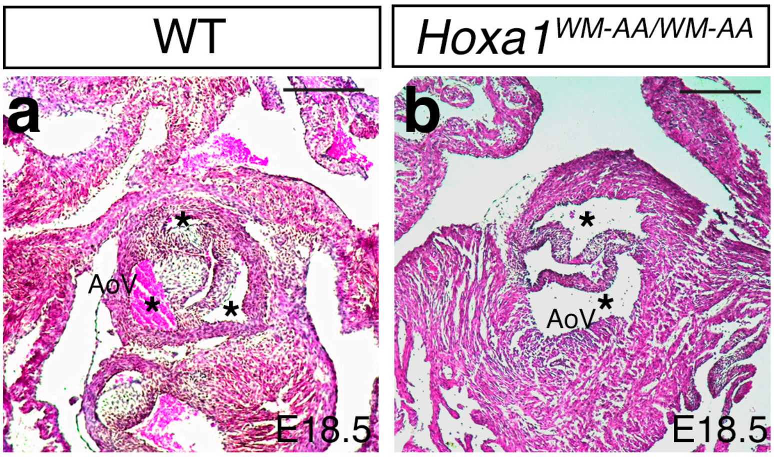

Supplementary Figure 7: *Hoxa1^{WM-AA}* knock-in mice display bicuspid aortic valve. **a**-**b**, Cross-sectional Hematoxylin and Eosin (H&E) images through the aortic valve of wild-type (WT; **a**) and *Hoxa1$^{WM-AA/WM-AA}$* (**b**) embryos at E18.5. Normal valve with three leaflets (asterisks) is observed in WT embryos (**a**), whereas bicuspid aortic valve is detected in the mutant (**b**). Data are representative of 52 wild-type and 5 *Hoxa1$^{WM-AA/WM-AA}$* embryos. Asterisks indicate the aortic sinus. Scale bars: 200 µm.

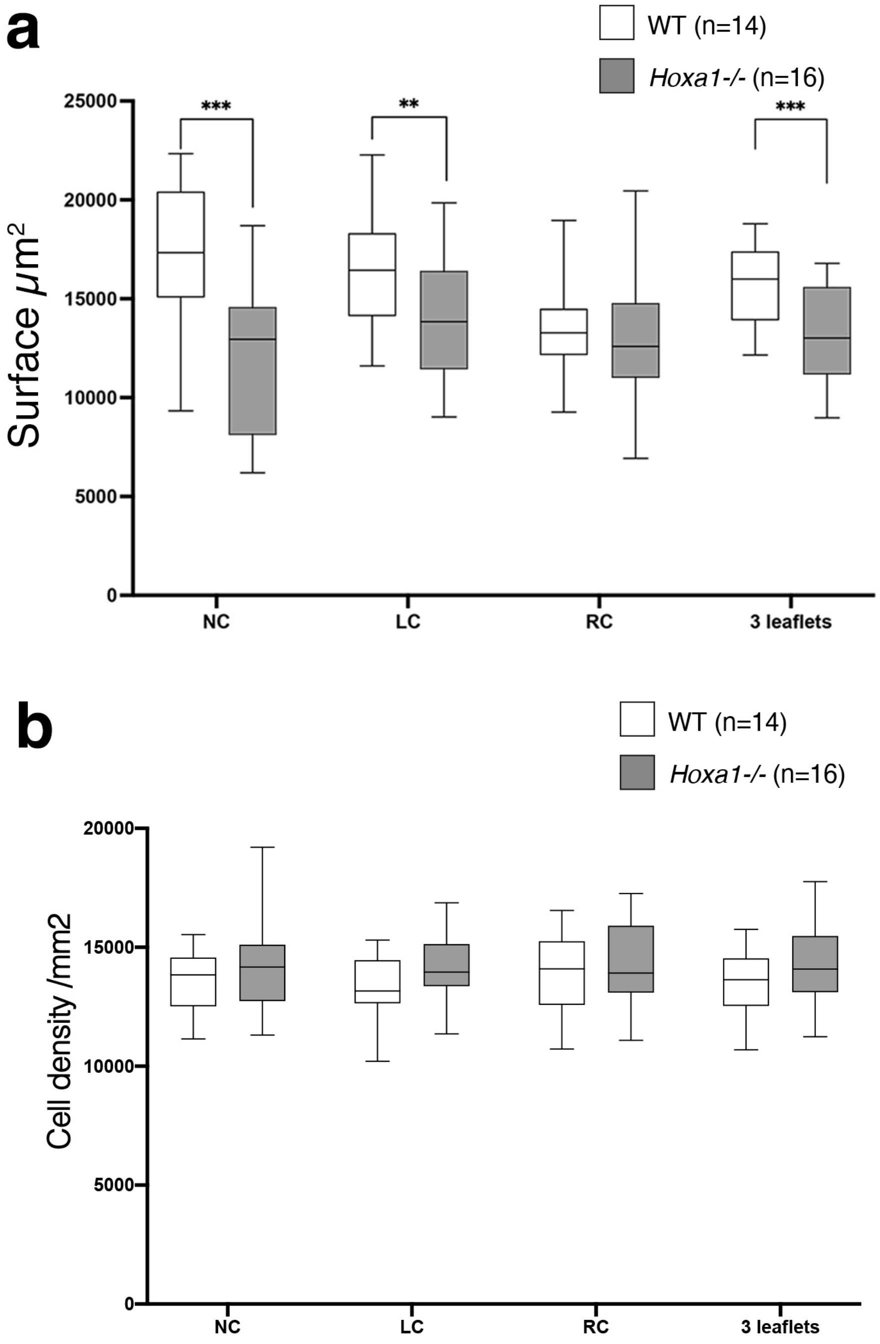

Supplementary Figure 8: Relative surface and cell density of the aortic valve leaflets. a, The relative surface of the three aortic valve leaflets was measured. There is a significant decrease in the *Hoxa1*$^{-/-}$ (Data are representative of 16 embryos) compared to the wild-type (WT; Data are representative of 14 embryos) littermates. **b**, The cell density was measured for each leaflet and no significant difference was observed between in the *Hoxa1*$^{-/-}$ (n=16 embryos) compared to WT (n=14 embryos) littermates. Data are shown as mean +/- SEM. Statistical values were obtained using the Mann-Whitney test. (***) significantly different $p<0.001$; (**) significantly different $p=0.02$; LC: left coronary leaflet; NC: non-coronary leaflet; RC: right coronary leaflet. Boxes and whiskers (min to max) show the values lower than the 25 percentile and greater than the 75 percentile. Source data are provided as a Source data file.

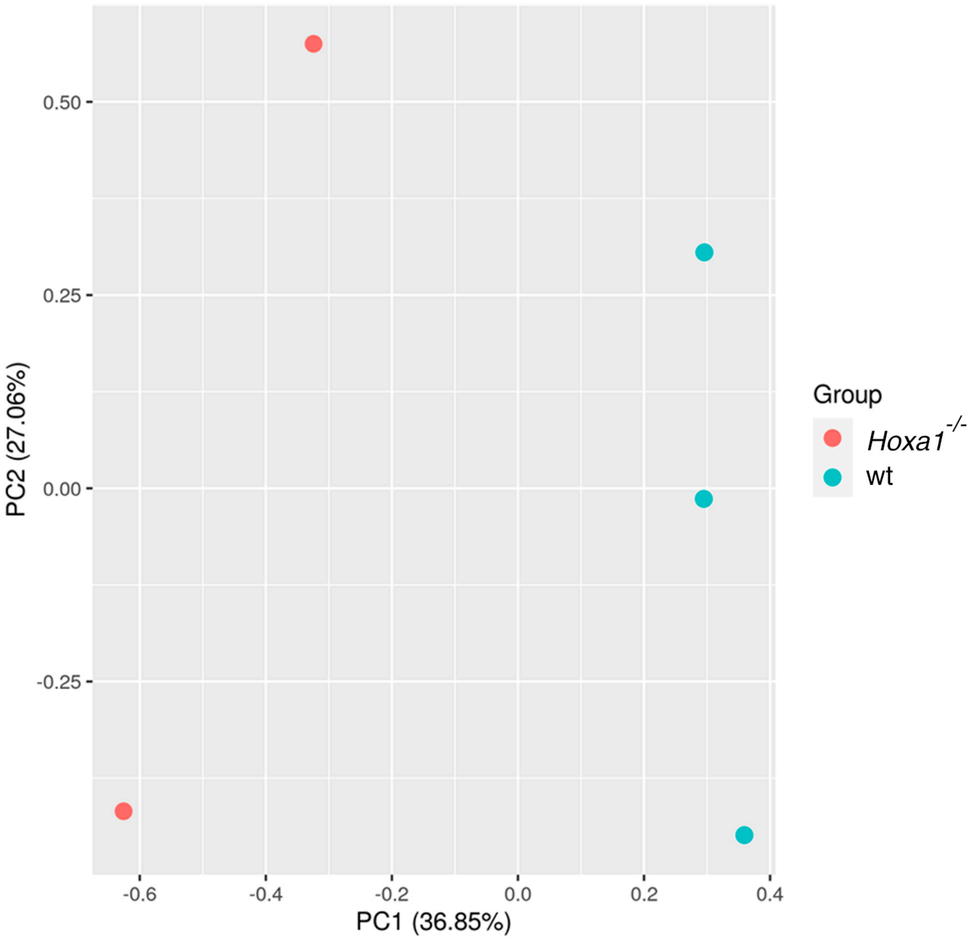

Supplementary Figure 9: Quality assessment of RNA-seq data performed with wild-type and *Hoxa1*$^{-/-}$ embryos. Principal component analysis (PCA) of RNA-sequencing datasets from wild-type (WT; blue) and *Hoxa1*$^{-/-}$ (red) embryos.

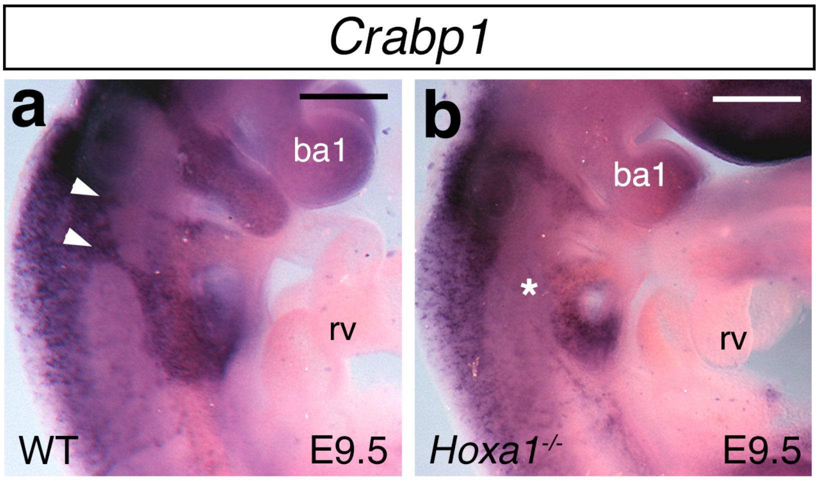

Supplementary Figure 10: Abnormal cardiac neural crest migration in *Hoxa1$^{-/-}$*. a,b Whole-mount *in situ* hybridization for *Crabp1* transcripts (marking neural crest cells) in wild-type (WT; **a**), and *Hoxa1$^{-/-}$* (**b**) embryos at E9.5. **a**, Arrowheads indicate normal post-otic streams of migrating neural crest cells. **b,** The asterisk indicates reduction of *Crabp1* expression in the 3rd pharyngeal arch of *Hoxa1$^{-/-}$* embryos compared to WT. ba: branchial arch; rv: right ventricle. Data are representative of 3 experiments. Scale bars: 200 µm.

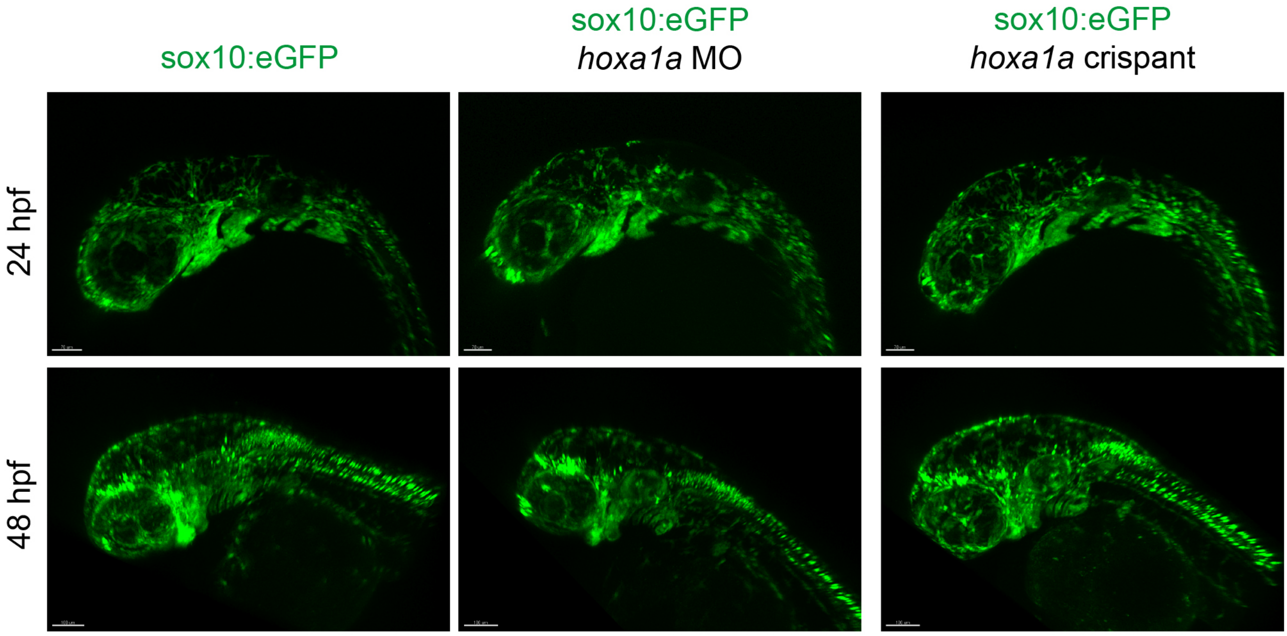

Supplementary Figure 11: GFP expression patterns in 24 and 48hpf *Tg(sox10:eGFP)* zebrafish. Representative maximal projections of non-injected fish, *hoxa1a* morphant (MO) and *hoxa1a* crispant are depicted. Data are representative of 3 experiments. Scale bars: top panel: 70 μm ; bottom panel : 100 μm.

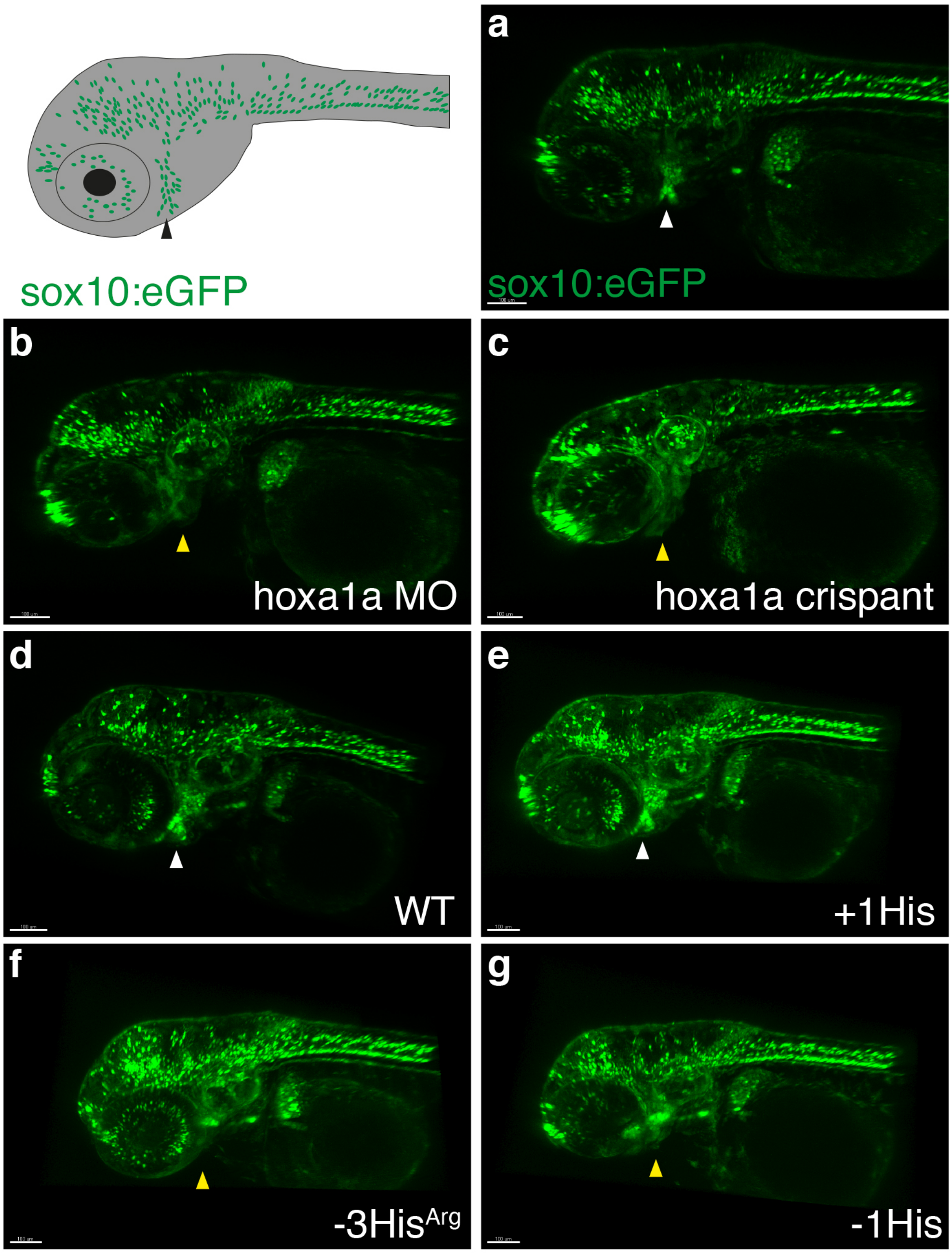

Supplementary Figure 12: GFP expression patterns in 3 dpf *Tg(sox10:eGFP)* zebrafish. Diagram depicts migrating neural crest cells. Representative maximal projections of (a) non-injected fish, (b) *hoxa1a* morphant (MO), (c) *hoxa1a* crispant, (d) fish injected with 25 pg of human WT mRNA, (e) fish injected with 25 pg of human +1His variant mRNA, (f) fish injected with 5 pg of human -3His^{Arg} variant mRNA, (g) fish injected with 5 pg of human -1His variant mRNA, are depicted. White and yellow arrowheads indicate the presence or the absence of GPF expression in the pharyngeal arch/OFT region respectively. Data are representative of 3 experiments.Scale bars: 100 µm.

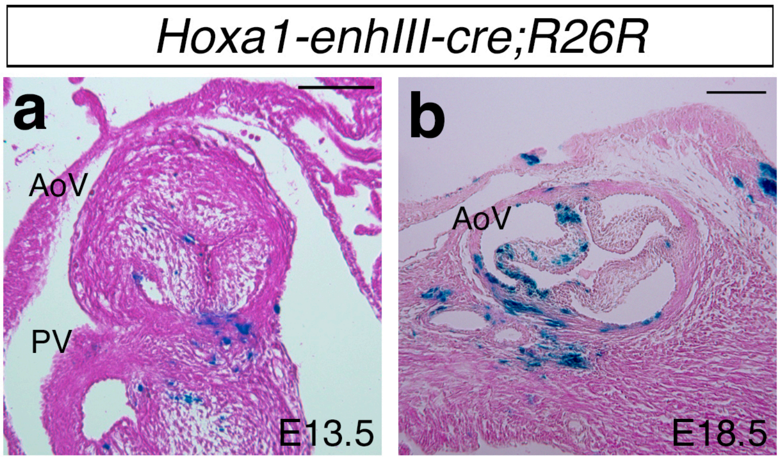

Supplementary Figure 13: Genetic lineage analysis of the *Hoxa1*-positive cells. **a-b**, *Hoxa1*-lineage visualized by X-gal staining of *Hoxa1-enhIII-cre;R26R-lacZ* embryos. **a**, Transversal section of the outflow tract at E13.5. β-galactosidase activity is observed in the outflow tract cushions. **b**, At E18.5, β-galactosidase activity is observed in cells forming the aortic valve leaflets. AoV, aortic valve; PV, pulmonary valve. Data are representative of 3 experiments. Scale bars: 100 µm.

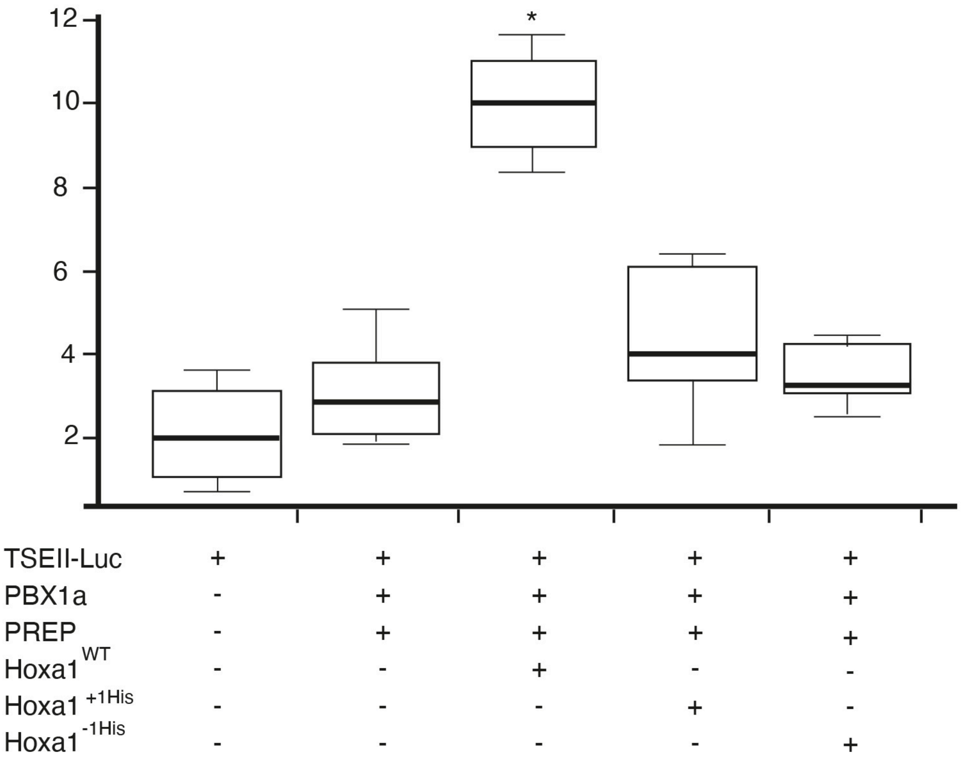

Supplementary Figure 14: Variations of the poly-histidine tract of Hoxa1 reduce transcriptional activity of the mouse protein. Luciferase assay showing transcriptional activity of wild-type (WT) and mutated Hoxa1 (+1His, and -1His). HEK293T cells were transfected with the TSEII-luciferase reporter construct, alone (TSEII-Luc) or together with expression plasmids for PREP1, PBX1a, and FLAG-Hoxa1 (WT, +1His, and -1His). Same quantity of DNA was transfected for each plasmid (n=6). *p=2.2 10^{-9}. Boxes and whiskers (min to max) show the values of lower and upper quartile. Statistical values were obtained using Pairwise t test with Holm P value adjustment method test. ‘n’ indicates number of biologically independent experiments (cell transfected independently in different week) in duplicate. Source data are provided as a Source data file.

Patient HOXA1 genotype	BAV Subtype	Predominant AV function	Leaflet calcification	Artery malformations	Eye-ear defects	Family history
BAV30 -1His, WT^A	Type 1, R-L	Moderate AR	-	N/A	N/A	no
BAV32 +1His^{Arg}, WT^A	Type 0	severe AR	-	N/A	N/A	no
BAV51 -1His, WT^A	Type 1, R-L	Severe AS	+	-	-	yes
BAV58 +1His, WT^A	Type 1, R-L	moderate AR	-	-	-	no
BAV89 -1His, WT^A	Type 1, R-L	Severe AS	+++	-	-	no
BAV103 -1His, WT^A	Type 1, R-L	Small AR	-	-	-	no
BAV116 +1His, WT^A	Type 1, R-L	moderate AR	-	-	-	no
BAV117 -1His, WT^A	Type 1, R-L	Severe AS	++	-	-	no
BAV121 -1His, WT^A	Type 0	Normal	-	-	-	no
BAV141 -3His^{Arg}, WT^A	Type 0	Severe AR	-	Coarctation	-	no
BAV148 -1His, WT^G	Type 0	Severe AR	-	-	-	no
BAV162 -1His, WT^A	Type 1, R-L	Severe AS	++	-	-	no
BAV164 -1His, WT^A	Type 0	Normal	-	-	-	no
BAV166 -3His^{Arg}, -3His^{Arg}	Type 0	Severe AS and Severe AR	++	-	-	no
BAV186 -1His, WT^G	Type 2	Moderate AS	++	-	-	no
BAV204 -1His, WT^A	Type 1, R-L	severe AS	++	-	-	no
BAV220 -1His, WT^A	Type 1, R-L	Normal	-	-	-	no
BAV240 -1His, WT^A	Type 1, R-L	Moderate AR	-	Cockett's syndrome	-	no
BAV277 -1His, WT^A	Type 1, R-L	Severe AS	++	-	-	no
BAV291 -1His, WT^A	Type 1, R-L	Severe AS	-	-	-	no
BAV292 -1His, WT^A	Type 1, R-L	Small AR	-	Iliac artery aneurysm	-	no
BAV302 -1His, WT^A	Type 1, R-L	Severe AS	++	-	-	no
BAV313 +1His, WT^A	Type 1, R-L	Severe AS and Moderate AR	++	-	-	no
BAV326 -1His, WT^A	Type 1, R-L	Severe AS	++	-	-	no
BAV330 -1His, WT^A	Type 1, R-L	Small AR	-	-	-	no
BAV347 +1His, WT^A	Type 1, R-NC	Normal	+	-	-	no
BAV378 -1His, WT^A	Type 1, R-L	Moderate AR	-	-	-	no
BAV386 -1His, WT^A	Type 1, R-L	Severe AR	-	-	-	no
BAV390 -1His, WT^A	Type 1, R-NC	Severe AR	-	-	-	yes

Supplementary Table 1: Clinical description of BAV patients with *HOXA1* variations.
Number of histidine in the poly-His tract is indicated according to genotypes described in **Table 2**. For example, WT^A= c.218G>A; WT^G= c.218G; -1His = c.[213_215delCCA;218G>A].
The BAV phenotype is described using the classification of Sievers (Sievers, H.-H. & Schmidtke, 2007). Type 0= no fusion; Type 1, R-L= fusion of the right and left coronary (R-L) leaflets; Type 1, R-NC= fusion of the right and non-coronary (R-NC) leaflets; AR, aortic regurgitation; AS, aortic stenosis; BAV, bicuspid aortic valve.

Variations	Impact on Histidine stretch	Short Names	Number of studied alleles	Frequencies in controls (%)
c.[213_215dupCCA;218G>A]	11His	+1His	3	0.18
c.[213_215dupCCA;218G]	9His1Arg1His	+1His^{Arg}	Not found in controls	
c.218G>A	10His	WT^{A}	1349	78.80
c.218G	8His1Arg1His	WT^{G}	300	17.52
c.[213_215delCCA;218G>A]	9His	-1His	51	2.98
c.[213_215delCCA;218G]	7His1Arg1His	-1His^{Arg}	Not found in controls	
c.[207_215delCCACCACCA;218G>A]	7His	-3His	Not found in controls	
c.[207_215delCCACCACCA;218G]	5His1Arg1His	-3His^{Arg}	2	0.12

Supplementary Table 2: Distribution of *HOXA1* Histidine stretch variations in the FranceGenRef panel (n=856 individuals).

Patient	Gene	RefSeq	cDNA change	Protein change	Exon n°	Genotype	rs ID	GnomAD_ Genome_AF	UMD score	UMD prediction
BAV121	*NOTCH1*	NM_017617	c.1945C>A	p.Pro649Thr	12	HTZ	rs780710009	0.00001	78	Pathogenic
BAV166	*NOTCH1*	NM_017617	c.368C>T	p.Thr123Met	3	HTZ	rs187473846	0.0005	41	Polymorphism
BAV166	*GATA5*	NM_080473	c.698T>C	p.Leu233Pro	3	HTZ	rs116164480	0.0009	59	Probably pathogenic

Supplementary Table 3: List of variants identified in this study. The Genome Aggregation Database (gnomAD): 123,136 exomes and 15,496 genomes; HTZ: heterozygous.

Genotype	Total mice at E18.5	Bicuspid Aortic Valve
Wild-type	10	0
Hoxa1$^{-/-}$	11	3
Wild-type	21	0
Hoxa1$^{-1His/-1His}$	23	4
Wild-type	65	0
Hoxa1$^{WM\text{-}AA/WM\text{-}AA}$	52	5

Supplementary Table 4: Table summarizing aortic valve defect in different genotypes.
