# Supplementary material for: Variations in the poly-histidine repeat motif of HOXA1 contribute to bicuspid aortic valve in mouse and zebrafish
Source: Nat Commun. 2023 Mar 20;14:1543. doi: 10.1038/s41467-023-37110-x (PMC10027860; doi:10.1038/s41467-023-37110-x)
Supplement: Supplementary file 3 — Description of Additional Supplementary Files [file 41467_2023_37110_MOESM3_ESM.pdf]

Description of Additional Supplementary Files

File Name: Supplementary Data 1
Description: List of genes significantly down-regulated and up-regulated in *Hoxa1* mutants (with log2(fold change) $p>0.2$ and FDR $p<0.05$) to perform GO analysis. The raw data for the transcriptomic data have been deposited in the Gene Expression Omnibus database from NCBI under accession code: GSE224217
