# Supplementary material for: Variations in the poly-histidine repeat motif of HOXA1 contribute to bicuspid aortic valve in mouse and zebrafish
Source: Nat Commun. 2023 Mar 20;14:1543. doi: 10.1038/s41467-023-37110-x (PMC10027860; doi:10.1038/s41467-023-37110-x)
Supplement: Supplementary file 5 — Reporting Summary [file 41467_2023_37110_MOESM5_ESM.pdf]

nature portfolio

Corresponding author(s): Stéphane ZAFFRAN

Last updated by author(s): Feb 13, 2023

Reporting Summary

Nature Portfolio wishes to improve the reproducibility of the work that we publish. This form provides structure for consistency and transparency in reporting. For further information on Nature Portfolio policies, see our Editorial Policies and the Editorial Policy Checklist.

Statistics

For all statistical analyses, confirm that the following items are present in the figure legend, table legend, main text, or Methods section.

n/a	Confirmed	
☐	☒	The exact sample size (*n*) for each experimental group/condition, given as a discrete number and unit of measurement
☐	☒	A statement on whether measurements were taken from distinct samples or whether the same sample was measured repeatedly
☐	☒	The statistical test(s) used AND whether they are one- or two-sided<br>*Only common tests should be described solely by name; describe more complex techniques in the Methods section.*
☒	☐	A description of all covariates tested
☒	☐	A description of any assumptions or corrections, such as tests of normality and adjustment for multiple comparisons
☐	☒	A full description of the statistical parameters including central tendency (e.g. means) or other basic estimates (e.g. regression coefficient) AND variation (e.g. standard deviation) or associated estimates of uncertainty (e.g. confidence intervals)
☐	☒	For null hypothesis testing, the test statistic (e.g. *F*, *t*, *r*) with confidence intervals, effect sizes, degrees of freedom and *P* value noted<br>*Give P values as exact values whenever suitable.*
☒	☐	For Bayesian analysis, information on the choice of priors and Markov chain Monte Carlo settings
☒	☐	For hierarchical and complex designs, identification of the appropriate level for tests and full reporting of outcomes
☒	☐	Estimates of effect sizes (e.g. Cohen's *d*, Pearson's *r*), indicating how they were calculated

Our web collection on statistics for biologists contains articles on many of the points above.

Software and code

Policy information about availability of computer code

Data collection	Microscopy images were acquired with ZEN 2011, and LAS software. DNA PCR were sequenced on automatic sequencer ABI 3130XL (Applied Biosystems). We used a custom java tool to genotype the interval of HOXA1 Histidin repeat sequence. The code for this tool is available at: https://github.com/lindenb/ScanHoxa1/ (https://doi.org/10.5281/zenodo.7625919).
Data analysis	3D images were performed using Fiji (imageJ 1.53q). Sequence analysis was performed using Seqscape software V5.2 (Applied Biosystems) and sequencher V5.4.6 (Gene Codes Corporation).<br>Data were processed with GraphPad Prism 9 and Microsoft excel 2016.<br>Exome sequence analysis was performed using UMD-predictor (v1.0.2), Qualimap (v2.2.2), VarAFT software (v2.17), STAR (v2.5.3a), Stringtie (v1.3.1c), DESeq2 (v1.34).

For manuscripts utilizing custom algorithms or software that are central to the research but not yet described in published literature, software must be made available to editors and reviewers. We strongly encourage code deposition in a community repository (e.g. GitHub). See the Nature Portfolio guidelines for submitting code & software for further information.

Data

Policy information about availability of data

All manuscripts must include a data availability statement. This statement should provide the following information, where applicable:

- Accession codes, unique identifiers, or web links for publicly available datasets
- A description of any restrictions on data availability
- For clinical datasets or third party data, please ensure that the statement adheres to our policy

Materials and raw data that support the findings are available upon request to the corresponding authors. Raw data are provided as a Source data file. The raw data for the transcriptomic data have been deposited in the Gene Expression Omnibus database from NCBI under accession code: GSE224217.

Human research participants

Policy information about studies involving human research participants and Sex and Gender in Research.

Reporting on sex and gender	Findings were not applied to one sex. Sex and gender were not considered in the design of this study.
Population characteristics	Population characteristics are provided in the manuscript. Age of patients at the diagnosis is provided in Supplementary Table 1.
Recruitment	Patients with BAV were recruited from La Timone Hospital, Marseille in strict compliance with all relevant ethical regulations. Patients with another clinical disorders such as autism or syndromic clinical presentations were excluded. Patients signed a letter of consent. All DNA and research protocols were collected in compliance with the Institutional Review Board after informed consent was obtained.
Ethics oversight	The study was approved by the Marseille ethic committee for the protection of the patient under the number #13.061

Note that full information on the approval of the study protocol must also be provided in the manuscript.

Field-specific reporting

Please select the one below that is the best fit for your research. If you are not sure, read the appropriate sections before making your selection.

☒ Life sciences ☐ Behavioural & social sciences ☐ Ecological, evolutionary & environmental sciences

For a reference copy of the document with all sections, see nature.com/documents/nr-reporting-summary-flat.pdf

Life sciences study design

All studies must disclose on these points even when the disclosure is negative.

Sample size	Sample sizes were chosen based on accepted standards in the field and previously published (PMID: 34648325, 34422841, or 32070236). For each quantification condition, >5 embryos were analyzed. No statistical method was used to predetermine the sample size.
Data exclusions	No data were excluded from analysis.
Replication	Reproducibility of findings were assessed via statistical tests, varying on sample size and experiment type. All the experiments were performed in at least 3 biologically independent embryos. All replicates reported in the manuscript and on which statistics are based are biological replicates. No technical replicates were used to calculate statistics. All attempts at replication of the results were successful.
Randomization	Each experiment contained animals from at least 2 different litters.
Blinding	Investigators were blinded to mouse or zebrafish genotypes during experiments, for performing sample analysis, imaging and quantification and genotyping was only performed at the end of the experiment.

Reporting for specific materials, systems and methods

We require information from authors about some types of materials, experimental systems and methods used in many studies. Here, indicate whether each material, system or method listed is relevant to your study. If you are not sure if a list item applies to your research, read the appropriate section before selecting a response.

Materials & experimental systems

n/a	Involved in the study
☐	☒ Antibodies
☐	☒ Eukaryotic cell lines
☒	☐ Palaeontology and archaeology
☐	☒ Animals and other organisms
☒	☐ Clinical data
☒	☐ Dual use research of concern

Methods

n/a	Involved in the study
☒	☐ ChIP-seq
☒	☐ Flow cytometry
☒	☐ MRI-based neuroimaging

Antibodies

Antibodies used

The following primary antibodies were used:
Mouse anti-FLAG (1:1,000; clone F2; cat: F1804; Sigma-Aldrich; Western-blot)
Mouse anti-β-ACTIN (1:5,000; clone AC-15; cat: A3854; Sigma; Aldrich; Western-blot)
Rat anti-PECAM/CD31 (1:100; cat: 553370; Pharmagen; Immunostaining)
Mouse anti-AP2-alpha (1:50; cat: 5E4; DSHB; immunostaining)

The secondary antibodies used are:
Donkey Anti-Rat Alexa 488 (1/500; cat: A21208, ThermoFisher Scientific)
Donkey Anti-Mouse Alexa 555 (1/500; cat: A31570, ThermoFisher Scientific)

Validation

Mouse anti-FLAG
validation for Western-blot: https://www.sigmaaldrich.com/FR/fr/tech-docs/paper/543711
Mouse anti-β-ACTIN
validation for Western-blot: https://www.sigmaaldrich.com/FR/fr/tech-docs/paper/382922
Rat anti-PECAM/CD31
validation from publication: Odelin et al. 2018 Development (PMID: 29158447)
Mouse anti-AP2-alpha
validation from publication: Roux et al. 2017 Mechanism of Development (PMID: 27956219)

Eukaryotic cell lines

Policy information about cell lines and Sex and Gender in Research

Cell line source(s)

HEK293T cell line was obtained commercially through ATCC (cat: CRL-3216)

Authentication

The cell line for this study was not authenticated.

Mycoplasma contamination

All cell lines have been tested negative for Mycoplasma contamination.

Commonly misidentified lines (See ICLAC register)

No commonly misidentified lines were used in this study.

Animals and other research organisms

Policy information about studies involving animals; ARRIVE guidelines recommended for reporting animal research, and Sex and Gender in Research

Laboratory animals

We used mouse (Mus musculus). Females between 8 to 30 weeks were used for breeding and dissected to collect embryos, 9 to 18 days after the observation of a vaginal plug. The following strains were used in this study:
Hoxa1neo mice were previously obtained from P. Chambon (Lufkin et al., 1991), Hoxa1WM-AA (Hoxa1tm1Rez; MGI:3056069), Hoxa1 KI mice were generated in a facility at the Université Catholique de Louvain, then imported in the local campus animal house, Hoxa1-enhIII-Cre (Bertrand et al., 2011), Wnt1-Cre (Tg(Wnt1-GAL4)11Rth; MGI:3524966), Tie2-Cre (Tg(Tek-cre)12Flv, MGI:2136412), Tnnt2-Cre (Tg(Tnnt2-cre)5Blh; MGI:2679081), RosaLacZ (Gt(ROSA)26Sor; MGI:1890203) and RosatdTomato (Gt(ROSA)26Sortm9(CAG-tdTomato)Hze; MGI:3809523) transgenic lines are available through the Jax Laboratory. CD1 and C57BL/6J mice were ordered from Charles River France.
Mice colonies were maintained in certified animal facilities in accordance with European guidelines, with a 7h to 19h light cycle. Embryos were collected 7 to 14 days after the observation of a vaginal plug. The room temperature ranged from 20 and 25°C. The relative ambient humidity at the level of mouse cages was 55 per cent +/-15. Each cage was provided with food, water and two types of nesting material.
We used zebrafish (Danio rerio) embryos (24-48hpf) and larvae (72 hpf; 7 dpf). The following strains were used in this study: AB wild-type (ABwt), sox10:eGFP (gift from Drs F. Djouad and D. Sapède, IRMB, Montpellier).

Wild animals

No wild animals were used in the study.

Reporting on sex

Sex was not considerated in this study.

Field-collected samples	No field collected samples were used in this study.
Ethics oversight	The experiments performed in SZ lab were approved by the local ethical committee under protocol N°32-08102012 and work in animal facility is approved by a National agrrement (C 13-013-08). The experiments performed in RR lab were approved by the local committee under protocols (agreement number LA1220028, project code 20280). The data obtained on zebrafish larvae were obtained in the animal facility of CJ lab under the agreement number B 34-172-41.

Note that full information on the approval of the study protocol must also be provided in the manuscript.
